# Obesity trends and risk factors in the South African adult population

**DOI:** 10.1186/s40608-015-0072-2

**Published:** 2015-10-13

**Authors:** Annibale Cois, Candy Day

**Affiliations:** Division of Epidemiology and Biostatistics, School of Public Health and Family Medicine, University of Cape Town, Anzio Road, Observatory, Cape Town, 7925 South Africa; Health Systems Trust, 34 Essex Terrace, Westville, Durban, 3630 South Africa

**Keywords:** Body mass index, Obesity, Sub-saharan Africa, Latent growth modelling

## Abstract

**Background:**

Obesity prevalence is increasing globally and contributes substantially to the burgeoning burden of non-communicable diseases. South Africa is particularly affected by this increasing trend and cross-sectional evidence suggests socioeconomic and behavioural variables as possible drivers. However, no large scale longitudinal study has attempted the direct identification of risk factors for progression towards obesity.

**Methods:**

This study analysed data on 10,100 South African adults (18 years and over) randomly selected in 2008 and successfully recontacted in 2010 and 2012. Latent Growth Modelling was used to estimate the average rate of change in body mass index (BMI) during the study period, and to identify baseline characteristics associated with different trajectories.

**Results:**

The overall rate of change in BMI during the study period was +1.57 kg/m^2^ per decade (95 % CI: 0.93 −2.22), and it was higher among women (+ 1.82 kg/m^2^ per decade, 95 % CI: 1.06 −2.58) than among men (+ 1.03 kg/m^2^ per decade; 95 % CI: 0.14 −1.93).

Female gender, younger age, larger waist circumference, white population group and higher household income per capita were baseline characteristics associated with higher rates of change.

The association between tobacco use and obesity was complex. Smoking was associated with greater waist circumference at baseline but lower rates of increase in BMI during the study period. Quitting smoking was an independent predictor of BMI increase among subjects with normal weight at baseline.

Among subjects with baseline BMI lower than 25 kg/m^2^, rates of changes were higher in rural than urban areas, and inversely related to the frequency of physical exercise.

**Conclusions:**

A strong positive trend in BMI remains in South Africa and obesity prevalence is likely to increase. Trends are not homogeneous, and high risk groups (subjects with high socioeconomic status, rural dwellers, young women) and modifiable risk factors (physical inactivity) can be targeted. Subjects quitting smoking should receive additional weight-loss support in order that the numerous health benefits of cessation are not reduced by increasing BMI. Centrally obese subjects should be targeted in campaigns.

**Electronic supplementary material:**

The online version of this article (doi:10.1186/s40608-015-0072-2) contains supplementary material, which is available to authorized users.

## Background

Little doubt exists of a worldwide rising trend in body mass index (BMI) and, consequently, in the prevalence of overweight and obesity.

The results of a large systematic review and meta-analysis of epidemiological studies carried out in 199 countries between 1980 and 2008, indicated that, in all but a few countries, the average age-standardised BMI of the adult population (20 years and older) increased during the period of observation. The average rate of increase was 0.4 kg/m^2^ per decade for men and 0.5 kg/m^2^ per decade for women [1].

The same review showed that the growing prevalence of obesity in the population, historically considered a problem of high-income countries, is increasingly affecting developing countries.

The Southern African region^1^ is particularly affected by this rising trend in BMI, and within this region South Africa — a middle income country undergoing rapid epidemiologic transition — shows an especially worrying picture. In 2008, the average BMI at population level was estimated at 26.9 kg/m^2^ among males (vs. a world average of 23.8 kg/m^2^), and 29.5 kg/m^2^ among females (vs. a world average of 24.1 kg/m^2^). The rate of growth, calculated over the period between 2000 and 2008, was 2.9 kg/m^2^ per decade for males and 1.6 kg/m^2^ per decade for females, with a remarkable increase compared to the preceding period 1980 to 2000, when the average rates of growth were 0.7 kg/m^2^ and 0.65 kg/m^2^ per decade, respectively among males and females [1].

These high values of BMI and seemingly increasing growth rates have an obvious correspondence with a large and rapidly increasing proportion of people overweight or obese. In the decade between 1998 and 2008, the estimated proportion of the South African adult population who was overweight or obese increased from 29.1 to 31.1 % among males (+6.9 % in relative terms), and from 56.2 to 59.5 % among women (+5.9 %) [2].

The reasons for these increasing trends are not completely understood.

A number of studies have gone beyond the mere estimation of the distribution of BMI in the population and prevalence of obesity, and analysed the association between obesity and a series of socio-economic and behavioural variables [3–11]. Replicating consistent research results from high-income countries, these (and other) studies have produced evidence of an association between BMI and gender (higher BMI among women than among men), alcohol use (positive relationship), tobacco (negative relationship), physical exercise (higher level of physical exercise associated with lower BMI), urban vs. rural living (with the former associated with higher values of BMI). The evidence that BMI is associated with socioeconomic status is also relatively established, but, unlike what has been observed in high-income countries, in South Africa, as in most low- and middle-income countries, high socioeconomic status has been associated with higher BMI [9, 11–13].

Considering the observed trends in the distribution of the above risk factors in the population (increasing urbanization and average socioeconomic status, stable but high alcohol consumption, decrease in smoking and level of physical exercise), [14–17] these findings support the hypothesis that the observed changes in BMI and prevalence of obesity in South Africa are at least partly driven by the changes in the distribution of the above risk factors.

However, all the cited studies refers to cross-sectional relationships between the variables of interest, while no study in South Africa — to our knowledge — has attempted the direct identification, using longitudinal data, of risk and protective factors for increasing BMI.

The aim of this study was to take advantage of longitudinal data collected by the Southern Africa Labour and Development Research Unit (SALDRU) during the first three waves of the National Income Dynamics Study (NIDS) to contribute to fill this knowledge gap and improve the understanding of the current trends in obesity in the South African population and its determinants.

The specific objectives of the study were: (1) to estimate the average rate of change in BMI between 2008 and 2012 in a representative sample of the South African adult population, and (2) to identify baseline socioeconomic and bio-behavioural characteristics of respondents predictive of different rates of changes in the follow-up period.

## Methods

The study analysed a subset of the data collected during the first three waves of the NIDS.

The NIDS survey method is described in detail by Woolard et al. [18]. The study was designed as a longitudinal panel survey of a nationally representative sample of households in South Africa. A two-stage cluster sampling design was used to randomly select about 7,300 households across 400 primary sampling units (areas), stratified by district council (a second level administrative division of South Africa’s territory). The first wave of the survey was conducted in 2008, and the target population was private households and residents in workers’ hostels, convents and monasteries, excluding other collective living quarters such as old age homes, hospitals, prisons and boarding schools. Trained fieldworkers were instructed to interview and collect anthropometric data on all available subjects belonging to the selected households. Written informed consent was obtained from participants. Written consent from a parent or legal guardian was obtained for those younger than 18 years.

The household level response rate at the first wave was 69 % and the individual response rate within households was 93 %. After the first successful contact, respondents were interviewed again in 2010 and 2012. Attrition rates were 19 % between wave 1 and wave 2, and 16 % between wave 2 and wave 3 [19].

The NIDS study, the dataset of which is publicly available for research purposes, has been granted ethical approval by the Commerce Faculty Ethics Committee at the University of Cape Town.

This present study considered the 10,100 individuals (out of an estimated South African adult population of 34 million) who were 18 years old and over at the time of the first interview, and who were successfully interviewed in the second and third wave. Data were extracted from wave 1 dataset Version 5.2, Wave 2 dataset Version 2.2, and Wave 3 dataset Version 1.2.

### Measures

#### Sociodemographic variables

Age in years was treated as a continuous variable. Education was measured in years of completed schooling and categorised as shown in Table 1. Individual monthly income was calculated as the summation of a wide array of sources, which is considered a more reliable method than the use of single questions [20]. Missing data in specific sources of income were imputed according to the procedure described by Argent [21]. Race was self-defined by participants according to the historical ‘population group’ categorization used in South Africa.^2^

**Table 1:** Sample descriptive statistics at baseline^a^

Variable	*N*	Median/percentage	IQR/frequency	Range
Men	10100	37.81 %	3819	
Age [years]	10099	38	[26 ; 52]	[18 ; 101]
Race	10100			
Black		81.07 %	8188	
Coloured		14.31 %	1445	
White		3.38 %	341	
Asian		1.25 %	126	
Education	10091			
None		15.61 %	1575	
Primary (1 – 7 years)		25.07 %	2530	
Secondary (8 – 12 years)		51.17 %	5164	
Tertiary (>12 years)		8.15 %	822	
Urban	10100	54.02 %	5456	
Quintile of household income per capita	10100			
I (0-217 ZAR^b^)		15.39 %	1554	
II (218 - 345 ZAR)		16.82 %	1669	
III (346 - 531 ZAR)		18.82 %	1901	
IV (532 - 992 ZAR)		22.84 %	2307	
V (993 - 62343 ZAR)		26.13 %	2639	
Urban	10100	54.02 %	5456	
Current smoking	9138	20.09 %	1845	
Current use of alcohol	9191	22.48 %	2066	
Exercise frequency	9159			
Low (<once a week)		79.40 %	7272	
Moderate (1/2 times a week)		9.95 %	911	
High (>2 times a week)		10.66 %	976	
Waist circumference [cm]	8376	85.55	[76.03 ; 98.05]	[30.40 ; 197.30]
BMI [kg/m^2^]	8177	25.18	[21.48 ; 30.85]	[10.61 ; 59.22]
BMI category	8177			
Underweight		5.38 %	440	
Normal weight		43.44 %	3552	
Overweight		23.08 %	1887	
Obese		28.10 %	2298	

^a^ *N* = number of non-missing values; IQR = interquartile range

Values are unweighted

^b^ZAR = South African Rand. 1 ZAR = 0.12 US Dollars (average exchange rate in 2008)

#### Body mass index and waist circumference

Duplicate measurements of height, weight and waist circumference were recorded. A third measure of weight and height was taken if their difference was greater than 1 kg and 0.5 cm, respectively. Implausible values and outliers (weight < 30 kg or > 250 kg, height < 60 cm or > 250 cm, waist circumference < 30 cm or > 200 cm) were excluded and considered as missing. The remaining values were averaged to generate a single value for each measurement. BMI was calculated by dividing weight in kg by the square of the height in meters, and categorised according to the World Health Organization’s cut-offs.^3^ The value of BMI was considered as missing for pregnant women.

#### Behavioural factors

Current smoking was assessed with a direct question (“*Do you smoke cigarettes?*”). Subjects were asked to indicate the frequency of alcohol use in a 8-points ordinal scale ranging from “*I have never drunk alcohol*” to “*Every day*”. Any response other than “*I have never drunk alcohol*” or “*I no longer drink alcohol*” was considered as indication of current alcohol use. A 5-points ordinal scale (from “*Never*” to “*Three or more times a week*”) was originally used to record the response of a single question on the frequency of physical exercise (“*How regularly do you exercise?*”). The five categories were collapsed into three for the purpose of these analyses, as shown in Table 1.

### Statistical analyses

Sample characteristics at baseline were described by median and interquartile range for continuous variables; and frequencies for categorical measures. The distribution of BMI in the sample, as well as weighted to refer to the whole population, was reported separately for each wave.

The average rate of change in BMI between 2008 and 2012 was estimated using a latent growth model, [22] in the whole sample, and separately for women and men. In this modelling framework, individual BMI at the three measurement occasions were used to estimate two factors which define the parameters of a growth line, namely its intercept and slope. The former represents the average value of BMI at baseline, while the latter captures the average rate of change during the study period.

To identify factors associated with different trajectories during the observation period, the slope and the intercept of the growth line were subsequently modelled as linear functions of the subjects’ baseline characteristics. Previous evidence indicates that trajectories of change in BMI may differ between individuals with low and high baseline BMI, [23] and to take into account this possibility we introduced among the predictors a binary variable categorising the subjects as normal/underweight vs. overweight/obese. Interaction terms between this variable and rural/urban dwelling and exercise frequency were also introduced in light of the results of a preliminary analysis, indicating the possibility of a different sign in the relationship between these risk factors and BMI trends among subjects with low and high BMI. Interaction terms with other behavioural predictors (smoking, alcohol) were tested, but did not produce significant changes in the model coefficients.

The model is conceptually equivalent to a two-level random coefficients model with measurements at level 1 and subjects at level 2, but requires less assumptions and allows for the direct assessment of the level of agreement with the data (model fit) [22, 24].

BMI values were adjusted for month of data collection in the light of previous evidence from population studies showing a significant (albeit small in magnitude) variation in BMI by season (with higher values recorded in winter than in summer), [25, 26] and the observation that the period of data collection differed remarkably across waves (see Additional file 1). Seasonal variation in BMI was modelled by a trigonometric spline with fixed period of 12 months and amplitude and phase estimated within the model [27].

Modelling details are provided as supplemental material (see Additional file 1).

In all analyses, the stratified, clustered, sampling scheme of the NIDS was taken into account by calculating standard error using the Huber-White sandwich estimator [28]. Sampling weights were used in the estimation to minimize the bias due to the unequal response rates in wave 1 across population strata, and to the non-random attrition between waves. Weights were incorporated into the analyses by weighting the likelihood functions according to the procedure described by Asparouhov (pseudo-likelihood method) [29].

Missing BMI values were accommodated using a Full Information Maximum Likelihood (FIML) estimator, under the hypothesis that values were missing at random conditional to the observed covariates.

Sensitivity analyses were conducted to evaluate (1) the sensitivity of the estimates to the implicit imputation procedure underlying the use of the FIML estimator and (2) the appropriateness of combining underweight with normal weight subjects in the analyses. In the first case the models were re-estimated including only respondents with valid BMI measurements in each wave, and in the second excluding underweight subjects.

The level of significance for statistical testing was fixed at the value *α*=0.05.

Statistical calculations were carried out using R Statistical Environment v. 3.0.2 (The R Foundation for Statistical Computing, Vienna, Austria; http://www.r-project.org) and Mplus v. 7.3 (Muthén & Muthén, Los Angeles, CA).

## Results

### Sample descriptive statistics

Sample descriptive statistics at baseline are depicted in Table 1. Additional file 1: Table S1 in the supplemental material shows the sample characteristics in the following waves.

The vast majority of participants were Black and Coloured, with the latter over-represented relative to the South African population. Conversely, Whites (and, to a lesser extent, Asians) were under-represented, owing to their lower response rate and higher between-wave attrition compared to the other population groups [19]. The average age of the subjects in the sample was 40.4 years (standard deviation, sd = 16.8 years), corresponding to an estimated average for the population of 39.2 years (standard error, se = 0.4 years). The distribution of household monthly income per capita was extremely skewed (which is a known characteristics of the unequal South African society) with only 21.3 % of the population (se = 1.96 %) above the average of 1829 ZAR.

Table 2 shows the sample and estimated population distributions of BMI in each wave.

**Table 2:** Sample distribution and population estimates of BMI in the three waves

Variable	Wave 1					Wave 2					Wave 3			
	Sample			Pop		Sample			Pop		Sample			Pop
		Mean/Perc		Mean/Perc			Mean/Perc		Mean/Perc			Mean/Perc		Mean/Perc
	*N*					*N*					*N*			
BMI [kg/m^2^]	8177	27.11		26.75		8184	27.70		27.36		8728	27.79		27.53
		(0.09)		(0.17)			(0.09)		(0.17)			(0.09)		(0.15)
BMI category														
Underweight	440	5.38 %		4.75 %		366	4.47 %		4.27 %		226	2.59 %		2.29 %
				(0.44 %)					(0.40 %)					(0.31 %)
Normal weight	3552	43.44 %		43.04 %		3073	37.55 %		39.11 %		3319	38.03 %		38.87 %
				(1.38 %)					(1.29 %)					(1.21 %)
Overweight	1887	23.08 %		24.84 %		2066	25.24 %		25.84 %		2403	27.53 %		27.86 %
				(1.01 %)					(0.89 %)					(1.00 %)
Obese	2298	28.10 %		27.37 %		2679	32.73 %		30.77 %		2780	31.85 %		30.98 %
				(0.91 %)					(1.06 %)					(0.95 %)

*N*= number of non-missing values

Values in brackets are standard deviations (sample) and standard errors (population)

### Average rate of change in BMI

The age-and-season adjusted average rate of change in BMI during the observation period was positive and statistically significant (+ 1.57 kg/m^2^ per decade, 95 % CI: 0.93 − 2.22). Disaggregating the analysis by gender produced an estimated rate of increase of 1.03 kg/m^2^ per decade (95 % CI: 0.14 − 1.93) among males, and 1.82 kg/m^2^ per decade (95 % CI: 1.06 − 2.58) among females.

### Factors associated with BMI trends

The estimated multivariate association between subjects’ baseline characteristics and the slope and the intercept of the growth line is illustrated in Table 3.

**Table 3:** Parameter estimates for the regression of the slope and intercept of the growth line on subjects’ baseline characteristics

	Slope [kg/m^2^ per decade]			Intercept [kg/m^2^]							
Independent variable	Coefficient	95 % CI		Coefficient	95 % CI						
Sex											
Male vs female	−*1.91*	(−2.93 ; −0.00)		−*1.54*	(−1.88 ; −1.21)						
Age											
10 year increase	−*0.90*	(−1.20 ; −0.50)		−0.03	(−0.15 ; 0.09)						
Race											
Coloured vs. Black	−0.51	(−1.98 ; 0.96)		−0.44	(−0.90 ; 0.03)						
White vs. Black	*2.58*	(0.46 ; 4.71)		−0.35	(−1.30 ; 0.60)						
Asian vs. Black	0.05	(−1.13 ; 1.0)		−*0.85*	(−1.36 ; -0.33)						
Education											
None vs. primary	−0.18	(−1.69 ; 1.34)		−0.27	(−0.75 ; 0.21)						
Secondary vs. primary	−0.05	(−1.13 ; 1.04)		0.17	(−0.19 ; 0.53)						
Tertiary vs. primary	0.67	(−0.82 ; 2.16)		0.17	(−0.38 ; 0.72)						
Income Quintile											
I vs. III	0.00	(−1.26 ; 1.26)		−0.18	(−0.60 ; 0.25)						
II vs. III	0.51	(−0.84 ; 1.86)		−0.16	(−0.58 ; 0.25)						
IV vs. III	1.28	(−0.08 ; 2.64)		−0.26	(−0.63 ; 0.10)						
V vs. III	*1.59*	(0.41 ; 2.78)		−0.21	(−0.58 ; 0.17)						
Residence											
Rural vs. urban	*1.16*	(0.02 ; 2.31)		−*0.44*	(−0.78 ; −0.09)						
Exercise Frequency											
Moderate vs. low	−0.14	(−1.50 ; 1.21)		0.35	(−0.04 ; 0.73)						
High vs. low	−*1.52*	(−2.78 ; −0.25)		*0.75*	(0.36 ; 1.15)						
Alcohol use	−0.14	(−1.10 ; 0.81)		−0.22	(−0.51 ; 0.07)						
Smoking	−*1.69*	(−3.00 ; −0.38)		−0.20	(−0.60 ; 0.21)						
Waist circumference											
10 cm increase	*0.70*	(0.20 ; 1.2)		*1.54*	(1.29 ; 1.79)						
BMI class											
Overweight/Obese vs.	−*7.99*	(−9.77 ; −6.21)		*7.23*	(6.58 ; 7.96)						
Normal/Underweight											
Rural x BMI class	−*1.98*	(−3.80 ; −0.16)		0.01	(−0.60 ; 0.58)						
Moderate Exercise x BMI class	0.92	(−1.35 ; 3.18)		−*1.10*	(−1.88 ; −0.33)						
High Exercise x BMI class	1.96	(−0.43 ; 4.35)		−*1.43*	(−2.22 ; −0.63)						

CI = Confidence Interval. Statistically significant coefficients in italic

As expected, the estimated baseline BMI was significantly higher in women than in men and positively associated with waist circumference. Also, it was remarkably lower among subjects in the Asian population group.

Rural dwellers had lower baseline BMI than urban dwellers, similarly among normal/underweight subjects (−0.44 kg/m^2^, 95 % CI: −0.78 ; −0.09) and among overweight/obese (−0.45 kg/m^2^, 95 % CI: −0.91 ; 0.01), but the difference was only significant in the former category.

High exercise frequency (more than 2 times a week) was associated with higher BMI, but only among normal/underweight subjects (0.75 kg/m^2^, 95 % CI: 0.36 ; 1.15).

Gender, age, race, income quintile, place of residence, smoking, waist circumference, BMI class and exercise frequency were associated with significantly different rates of change in BMI during the observation period.

Among sociodemographic characteristics, female gender, younger age, being White rather than Black and belonging to the highest quintile of the income distribution were independent predictors of higher rates of increase.

Among behavioural characteristics, smoking was significantly associated with lower rates of increase. To further explore this relationship, we re-estimated the model adding as additional predictors two dummy variables indicating smoking cessation (among baseline smokers) and smoking inception (among baseline abstainers) during the study period, and their interaction with BMI class.

The results showed that smoking cessation was associated with significantly higher rates of change in BMI among normal/underweight subjects (3.56 kg/m^2^ per decade, 95 % CI: 1.49 ; 5.63), but it did not affect the trend among overweight/obese. Conversely, smoking inception was associated with lower rates of change, but only among overweight/obese subjects (−4.47 kg/m^2^ per decade, 95 % CI: −7.69 ; −1.24).

Exercising more than twice a week at baseline predicted lower rates of changes among normal/underweight subjects (−1.52 kg/m^2^ per decade, 95 % CI: −2.78 ; −0.25).

Waist circumference was independently associated with higher rates of increase, while belonging to the overweight/obese class at baseline predicted much lower rates of increase.

Finally, rural residence predicted higher rates of increase among normal/underweight subjects (+ 1.16 kg/m^2^ per decade, 95 % CI: 0.02 ; 2.31), but was negatively, not significantly correlated with the slope among overweight/obese subjects (−0.82 kg/m^2^ per decade, 95 % CI: −2.19 ; 0.55).

Repeating the estimation (1) excluding the 988 subjects with missing values for BMI on one or more measurement occasions and (2) the 440 respondents classified as underweight at baseline did not change the overall pattern of association and the order of magnitude of the regression coefficients (see Additional file 1: Table S2 in the supplemental material).

### High-risk subpopulations

The simultaneous presence of specific combinations of risk factors makes some population strata at especially high risk of rapidly increasing BMI and movement from the normal BMI range towards overweight or obesity.

**Fig. 1:**
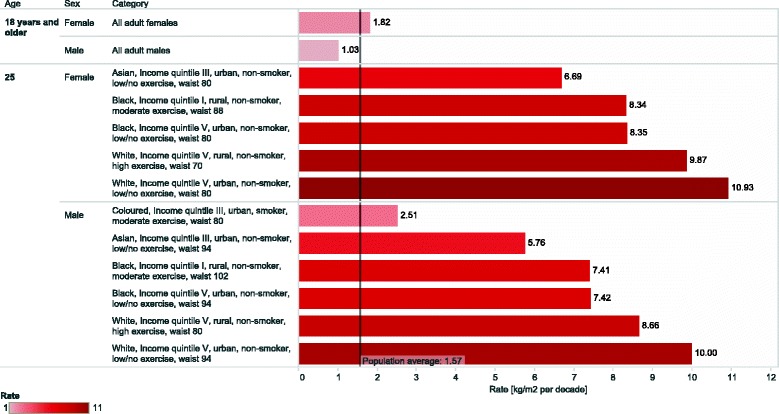
Estimated rate of increase in BMI in selected high-risk sub-groups of the South African adult population between 2008 and 2012. Values represent the average rate of increase in BMI for individuals with normal weight at baseline and the combination of sociodemographic and bio-behavioural characteristics indicated in the figure, estimated form the multivariate model described in the text. The values of the variables not explicitly indicated in the figure are set to the population average

Figure 1 compares the average rates of change in BMI estimated from the model for individuals with normal BMI and selected combinations of sociodemographic and bio-behavioural characteristics at baseline. Selections include one or more high risk characteristics for each gender and race group, with waist circumference below-, at or above the World Health Organization’s cut-offs indicating increased cardiovascular risk [30].

The purpose of the figure is illustrative, without any claim of completeness. It aims at highlighting some sub-groups of the South African population which showed, according to our model, rates of increase in BMI during the study period well above the country average.

## Discussion

From a public health perspective, the first consideration we can draw from the analyses above is that the strong positive trend in BMI observed in the last decades in all countries of Southern Africa is still present in the South African population. Even considering the reduction in the rate of increase observed in men, this fact in itself calls for the urgent implementation of public health interventions to curb the obesity epidemic. In the absence of effective interventions, the overall proportion of adult South Africans who are overweight or obese is extremely likely to increase by a few percentage points by 2020, rather than to decrease by 10 % as per South African Government’s strategic plan [31]. This is likely to further increase rather than reduce the relative mortality from non-communicable diseases, which have become the largest broad cause of premature mortality since around 2009 [32].

A second consideration that can be drawn from analyses suggests that trends in obesity are not homogeneous across population strata defined by biological, behavioural and socioeconomic characteristics. The identification of risk factors in the latter two categories has immediate potential from a public health perspective, because these factors are potentially modifiable. The identification of biological factors is also of public health interest since this knowledge can help targeting high risk groups more effectively, avoiding the waste of resources associated with interventions excessively broad in scope.

The existence of socioeconomic inequalities in obesity prevalence is a well-established finding, and has been previously confirmed in the South African population [3, 8]. This study adds to those findings showing that socioeconomic inequalities exist also in the temporal trends of obesity. In particular, our analysis suggests that subjects at the extreme of the income distribution (fourth and fifth quintiles) and belonging to the White population group (a strong indicator of high socioeconomic status) are more at risk of increasing their BMI compared to those in the lower socioeconomic strata. This conclusion is supported by the fact that subjects with tertiary education (another strong indicator of high socieconomic status) also show higher rates of change, even though the relationship does not reach statistical significance.

Our results also indicate rural vs. urban dwelling as a risk factor for increasing BMI. This is an interesting finding, because the results from other studies show that the prevalence of obesity in South Africa is higher in urban that rural areas, especially among women [3]. Our analyses confirm that rural dwelling is associated with significantly lower baseline BMI, but also suggest that subjects in rural areas are ‘catching up’, possibly because of the rapid spread of urban lifestyles (high consumption of processed food, reduced physical activity) into rural areas. From a public health perspective, this indicates that targeting rural areas before the prevalence of overweight and obesity increases to ‘urban’ level, could have a significant impact on the future trends in the obesity epidemic in the country.

BMI in women is increasing more rapidly than in men. Moreover, while the rate of increase seems to be slowing down among men, among women there is no evidence of a similar reduction. Young women, in particular, seem to be the most vulnerable.

Among behavioural factors, our study offers evidence that smokers tend to increase their BMI less than non-smokers, that smoking cessation is an independent risk factor for faster BMI increase among subjects with normal weight, and that obese/overweight subjects who start smoking tend to lose weight.

However, these results (which add to the consistent findings of many population studies showing that smoking is associated with lower BMI, [33] and that smoking cessation is associated with weight gain [34]) should not be interpreted as a support of smoking as an efficient way of controlling body weight. The relationship between smoking and body weight is incompletely understood and, even though a direct effect of nicotine on body weight is plausible (because nicotine increases energy expenditure and could reduce appetite), many other factors are likely to be involved in explaining the observed relationships. Among those, the results of various studies suggest that smoking inception is more frequent among subjects with greater weight concern and previous attempts to lose weight, which may indicate that changes in body weight following smoke inception are at least partly determined by psychological and behavioural factors that precede the initiation [35]. These factors were not measured in our study.

Moreover, a growing literature shows that smoking is associated with increased insulin resistance and risk of type 2 diabetes, as well as greater waist circumference (an indicator of the amount of visceral adipose tissue) thus suggesting that, despite the lower BMI, smokers, and especially heavy smokers, have an increased cardiovascular risk compared to non smokers [35]. The finding of a greater waist circumference among smokers is supported by the results of a secondary cross-sectional analysis of our data, which show that heavy smoking (>20 cigarettes/day) is significantly associated with greater waist circumference compared to no smoking (linear regression coefficient c = 3.25 cm, 95 % CI: 0.25 ; 6.2)^4^.

In any case—regardless of the dubious underlying causal mechanism—our finding that smoking cessation is associated with weight gain might be of public health interest because of the observed downward trend in the number of smokers in South Africa, [16] which, besides its overwhelming benefits for general population health, could foster an increase in BMI. Subjects who decide to quit smoking should be considered for obesity prevention strategies.

Among normal/underweight subjects, high levels of physical exercise were associated with lower rates of increase of BMI during the study period, but with higher baseline BMI. A possible explanation of this incongruence between cross-sectional and longitudinal relationships is that they might be expression of reversed causal processes. That is, while the causal precedence in the longitudinal relationship between exercise frequency and subsequent BMI change is determined by the temporal sequence, it might be that the observed cross-sectional association is the result of a greater tendency of subjects with higher BMI to exercise more in order to decrease their weight.

Finally, special consideration of waist circumference is deserved. In our analysis, waist circumference (besides being a risk factor for cardiovascular disease *per se*) was directly associated with higher rates of increase in BMI, independently of the BMI class. These considerations strongly suggest centrally obese subjects as primary targets of obesity reduction campaigns.

Several limitations of this study need to be acknowledged. Low reliability of self-report data, including those on physical exercise, alcohol and tobacco use is a well-known problem in population-based surveys and the measurements used in this study are no exception. However, in absence of specific reasons to think of an association between measurement error and individual BMI, it is probable that this measurement unreliability resulted in observed associations biased towards the null [36]. More precise measurements are therefore likely to strengthen the result of our analyses rather than invalidate them.

Independent variables introduced in the models as possible predictors of different BMI trajectories were identified according to previous evidence of association with BMI in population studies in SubSaharan Africa and availability in the NIDS dataset. Other factors are likely to play a significant role in explaining inter-individual differences, and, among those it is worth mentioning dietary habits and metabolic disorders, especially diabetes. For both these variables, the NIDS dataset provides neither direct measures nor reliable proxies^5^.

Suboptimal response and greater attrition rates were observed in some social strata in the NIDS survey. Even though differences between respondents and non-respondents in observed characteristics have been taken into account through appropriate adjustment of sampling weights, we cannot exclude the possibility that unobserved differences might have biased the results of our study in an unpredictable way.

Finally, the availability of three successive measurements only allowed for the estimation of linear trends. The availability of measurements from the forthcoming waves of the NIDS study will allow the possibility of non-linear trends and to better forecast future scenarios.

## Conclusion

To our knowledge, this is the first study to attempt the direct identification of risk and protective factors for increasing BMI in the South African population using longitudinal rather than cross-sectional data.

The results of the analyses indicate that the positive trend in BMI observed in the last decades in all countries of Southern Africa is still strongly present in the South African population, especially among women.

Trends are not homogeneous across population strata, and public health interventions can achieve a more efficient use of the available resources by targeting high risk groups (subjects with high socioeconomic status, rural dwellers, young women and those who are centrally obese) and modifiable risk factors associated with increased risk of weight gain (physical inactivity). The finding that the BMI of normal weight subjects in rural areas (where the prevalence of obesity is currently lower) is increasing at a faster pace than in those living in cities is of particular interest from a public health perspective. It calls for the urgent implementation of preventive interventions in rural environments, before the prevalence of overweight and obesity increases to ‘urban’ levels, with a significant negative impact on the future trends in the obesity epidemic in the country.

Finally, the complex relationship between smoking, smoking cessation and body weight, combined with the observed downward trend in the prevalence of smokers in South Africa, calls for providing subjects quitting smoking with additional weight-loss support in order that the numerous health benefits of cessation are not reduced by increasing BMI.

## Endnotes

^1^ Including Botswana, Lesotho, Namibia, South Africa and Zimbabwe.

^2^ Under apartheid, South Africans were categorised into one of four socially defined groups: Asian (or Indian), Black (or African), Coloured (wide grouping of people of mixed ancestry) and White (or European). In this sense, race is closely and enduringly correlated with socioeconomic status.

^3^ Underweight (BMI < 18 kg/m^2^); Normal weight (18 kg/m^2^ ≤ BMI < 25 kg/m^2^); Overweight (25 kg/m^2^ ≤ BMI < 30 kg/m^2^); and Obese (BMI ≥ 30 kg/m^2^) [30].

^4^ The estimate is adjusted for BMI, sex, age, education, urban/rural dwelling and alcohol use, and refers to the sample at baseline.

^5^ The NIDS dataset only includes indicators of expenditure per different types of food at household level, but no information on individual food intake. Presence/absence of diabetes is included as self-report (“*have you even been diagnosed with diabetes?*”), and a preliminary analysis of the between-wave congruence of these measures suggested very low reliability [37].

## Additional file

Additional file 1
**Statistical analyses and further results.** Methodological details on statistical analyses. Further results. Portable Document Format (.pdf) file. (PDF 214 kb)

## Abbreviations

BMI: Body mass index
SALDRU: Southern Africa labour and development research unit
FIML: Full information maximum likelihood
NIDS: National income dynamics study
