# Supplementary material for: Obesity trends and risk factors in the South African adult population
Source: BMC Obes. 2015 Oct 13;2:42. doi: 10.1186/s40608-015-0072-2 (PMC4603579; doi:10.1186/s40608-015-0072-2)
Supplement: Additional file 1 — Statistical analyses and further results. Methodological details on statistical analyses. Further results. Portable Document Format (.pdf) file. (PDF 214 kb) [file 40608_2015_72_MOESM1_ESM.pdf]

Additional File 1

Obesity trends and risk factors in the South African adult population

Annibale Cois[1,2], Candy Day[2]

[1] Division of Epidemiology and Biostatistics
School of Public Health and Family Medicine
University of Cape Town, Observatory 7925
Cape Town, South Africa

[2] Health Systems Trust
34 Essex Terrace, Westville 3630
Durban, South Africa

Table of Contents:

Weighted distribution of sample characteristics 2
Adjustment for period of data collection 2
Latent Growth Model: Mplus code and model results 4
Sensitivity analyses 7
References 9

Weighted distribution of sample characteristics

Table A1 depicts the characteristics of the sample at wave 1, weighted to refer to the South African adult population in 2008.

Table S1: Weighted sample descriptive statistics at baseline[a]

Variable	Weighted Median/Percentage
**Men**	43.65%
**Age [years]**	36
**Race**	
Black	81.03%
Coloured	7.86%
White	8.74%
Asian	2.35%
**Education**	
None	10.91%
Primary	20.49%
Secondary	56.27%
Tertiary	12.32%
**Urban**	59.66%
**Household income per capita [ZAR]**	642
**Current smoking**	20.27%
**Current use of alcohol**	25.88%
**Exercise frequency**	
Low (<once a week)	73.69%
Moderate (1/2 times a week)	13.13%
High (>2 times a week)	13.17%
**Waist circumference [cm]**	85.55
**BMI [kg/m^2]**	25.12
**BMI category**	
Underweight	4.89%
Normal weight	44.06%
Overweight	24.26%
Obese	26.79%

[a] ZAR = South African Rand.

Adjustment for period of data collection

Figure A1 shows the distribution by month of the absolute number of interviews carried out in each wave of the NIDS.

The distribution is quite different in the three waves. In particular, in wave 1 most of the data collection was carried out between February and June, in months characterised by warm temperatures, which have been associated to lower values on BMI.[1, 2] Conversely, in wave 2 and wave 3, most of data collection was carried out in winter, when BMI tends to increase.

Neglecting these differences might have biased the results of the trend estimation, most probably upwards. In fact, part of the observed positive difference between the average BMI at wave 1 and the average BMI at wave 2 and 3 can be explained by the seasonal effect (increased BMI in cold months compared to warm months) and not attributable to 'true' changes in the population BMI.

Figure S1: Number of subjects interviewed in the three waves of NIDS, by month

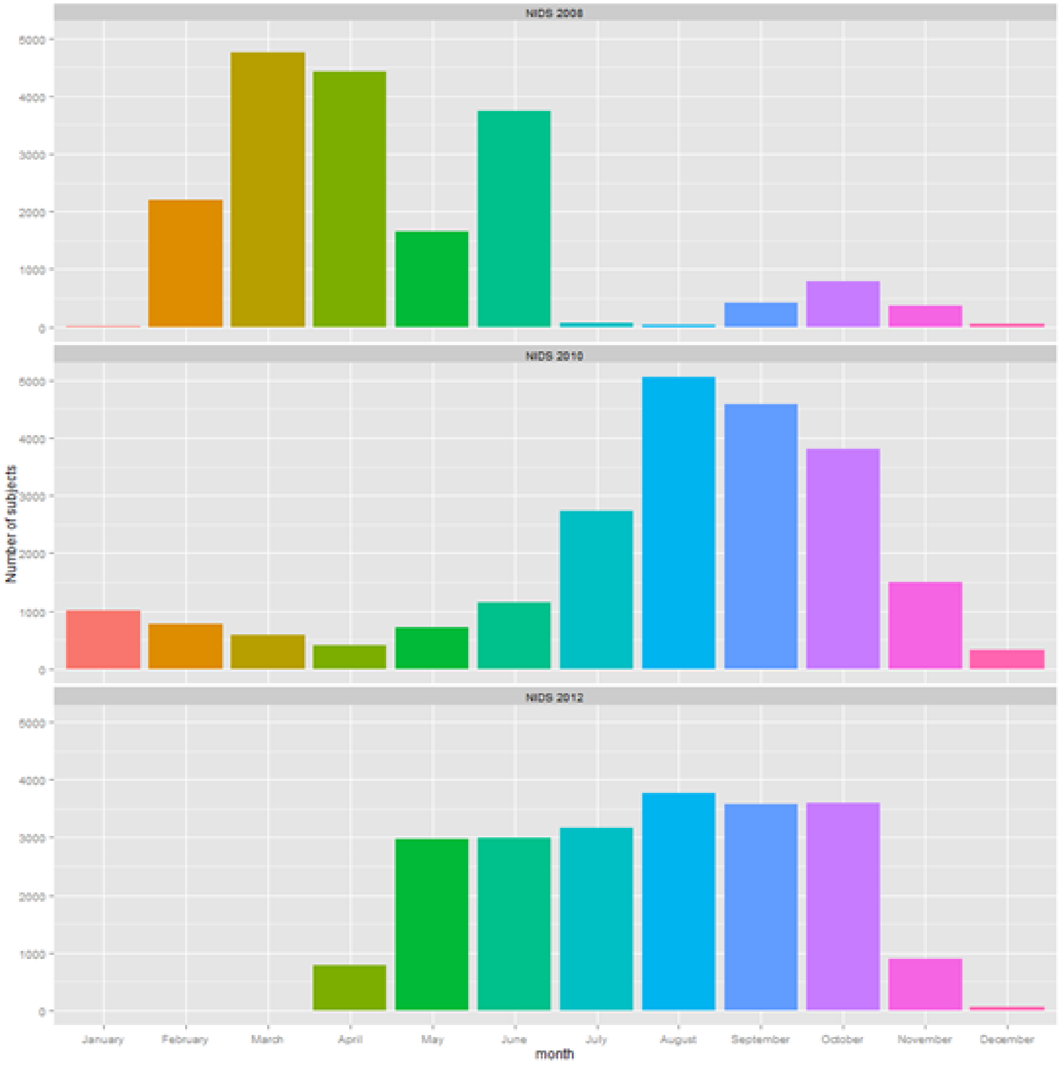

Adjustment for seasonal effect was done introducing in the latent growth models, as time

dependent covariates, two new variables, S_1 and S_2, defined as follows:

$$S_1 = \sin(m\frac{2\pi}{12}) \qquad S_2 = \cos(m\frac{2\pi}{12})$$

where m is the integer number representing the month of measurement (1-12).

The estimated coefficients and of S_1 and S_2, taken together, allowed for the identification of the best-fitting sinusoidal curve representing the seasonal variation of BMI in the considered population. The coefficient were constrained to be equal across waves, which corresponds to the assumption of constant seasonal effect.

To check the plausibility of the assumptions underlying this procedure, we calculated amplitude A and phase Φ of the seasonal effect, that is the maximum difference in BMI due to season and the position of the maximum during the year. If β_1 and β_2 are the coefficients of S_1 and S_2, respectively, it can be shown that:[3]

$$A = 2\sqrt{\beta_1^2 + \beta_2^2} \qquad \Phi = \arctan(\frac{\beta_2}{\beta_1})$$

The estimated seasonal effect had an amplitude of 0.20 Kg/m^2 and the maximum occurred in August, during the southern hemisphere winter. Both values are plausible, and in line with with the results of other large population studies.[1]

Latent Growth Model: Mplus code and model results

Code (extract):

```
VARIABLES = bmi1 bmi2 bmi3                   ! BMI at wave 1,2 and 3
            sin1 cos1 sin2 cos2 sin3 cos3    ! Trigonometric splines elements
            sex                              ! Gender
            age                              ! Age
            race_2 race_3 race_4             ! Dummy vars for race
            educat_1 educat_3 educat_4       ! Dummy vars for education
            incq_1 incq_2 incq_4 incq_5      ! Dummy vars for income quint.
            rural                            ! Rural vs urban
            EXMOD EXHIG                      ! Dummy vars for exercise freq.
            alc                              ! Alcohol use
            smok                             ! Smoking
            waist                            ! Waist circumference
            OVERWGT                          ! Overweight/obese vs. normal/underweight
            ORUR                             ! Interaction OVERWGT x rural
            OEXMOD OEXHIG                    ! Interaction OVERWGT x EXMOD,EXHIG
            ;

MODEL:

INT SLO | bmi1@0 bmi2@2 bmi3@4;              ! Latent INTercept and SLOpe
```

```
  bmi1 ON ssin1 scos1 (1-2);
  bmi2 ON ssin2 scos2 (1-2);
  bmi3 ON ssin3 scos3 (1-2);

  SLO INT  ON
           sex age
           race_2 race_3 race_4
           educat_1 educat_3 educat_4
           incq_1 incq_2 incq_4 incq_5
           rural
           EXMOD EXHIG
           alc smok
           waist
           OVERWGT
           ORUR
           OEXMOD OEXHIG
          ;

  SLO with INT;
```

Further indices of model fit and coefficient estimates:

```
Chi-Square Test of Model Fit
          Value                            146.899
          Degrees of Freedom                    39
          P-Value                           0.0000
          Scaling Correction Factor         2.3051

Chi-Square Test of Model Fit for the Baseline Model
          Value                           7636.341
          Degrees of Freedom                    87
          P-Value                           0.0000

RMSEA (Root Mean Square Error Of Approximation)
          Estimate                           0.018
          90 Percent C.I.                    0.015  0.022
          Probability RMSEA <= .05           1.000
CFI/TLI
          CFI                                0.986
          TLI                                0.968

SRMR (Standardized Root Mean Square Residual)
          Value                              0.012

MODEL RESULTS
                                                      Two-Tailed
                    Estimate       S.E.  Est./S.E.    P-Value


 INT      |
    BMI1               1.000      0.000    999.000    999.000
```

```
    BMI2               1.000      0.000    999.000    999.000
    BMI3               1.000      0.000    999.000    999.000

 SLO      |
    BMI1               0.000      0.000    999.000    999.000
    BMI2               2.000      0.000    999.000    999.000
    BMI3               4.000      0.000    999.000    999.000

 SLO      ON
    SEX               -0.191      0.052     -3.635      0.000
    AGEB              -0.009      0.002     -5.129      0.000
    RACE_2            -0.051      0.075     -0.677      0.498
    RACE_3            -0.007      0.086     -0.081      0.936
    RACE_4             0.258      0.108      2.383      0.017
    EDUCAT_1          -0.018      0.077     -0.227      0.821
    EDUCAT_3          -0.005      0.055     -0.085      0.932
    EDUCAT_4           0.067      0.076      0.878      0.380
    INCQ_1             0.000      0.064      0.000      1.000
    INCQ_2             0.051      0.069      0.745      0.456
    INCQ_4             0.128      0.069      1.843      0.065
    INCQ_5             0.159      0.060      2.632      0.008
    RURAL              0.116      0.059      1.986      0.047
    EXMOD             -0.014      0.069     -0.209      0.834
    EXHIG             -0.152      0.065     -2.350      0.019
    ALC               -0.014      0.048     -0.283      0.777
    SMOK              -0.169      0.067     -2.529      0.011
    WAIST              0.007      0.002      2.985      0.003
    OVERWGT           -0.799      0.091     -8.807      0.000
    ORUR              -0.198      0.093     -2.135      0.033
    OEXMOD             0.092      0.116      0.795      0.426
    OEXHIG             0.196      0.122      1.610      0.107

 INT      ON
    SEX               -1.544      0.172     -8.974      0.000
    AGEB              -0.003      0.006     -0.433      0.665
    RACE_2            -0.434      0.235     -1.846      0.065
    RACE_3            -0.848      0.264     -3.215      0.001
    RACE_4            -0.349      0.485     -0.721      0.471
    EDUCAT_1          -0.272      0.245     -1.109      0.268
    EDUCAT_3           0.167      0.184      0.909      0.363
    EDUCAT_4           0.170      0.281      0.607      0.544
    INCQ_1            -0.176      0.217     -0.811      0.417
    INCQ_2            -0.164      0.210     -0.780      0.436
    INCQ_4            -0.263      0.185     -1.418      0.156
    INCQ_5            -0.206      0.193     -1.068      0.285
    RURAL             -0.436      0.176     -2.476      0.013
    EXMOD              0.346      0.195      1.774      0.076
    EXHIG              0.753      0.201      3.744      0.000
    ALC               -0.222      0.146     -1.515      0.130
    SMOK              -0.195      0.208     -0.939      0.348
    WAIST              0.154      0.013     12.065      0.000
    OVERWGT            7.273      0.349     20.834      0.000
    ORUR              -0.014      0.298     -0.045      0.964
```

```
    OEXMOD            -1.106      0.395     -2.797      0.005
    OEXHIG            -1.426      0.407     -3.504      0.000

 BMI1     ON
    SSIN1             -0.038      0.071     -0.531      0.595
    SCOS1             -0.090      0.087     -1.032      0.302

 BMI2     ON
    SSIN2             -0.038      0.071     -0.531      0.595
    SCOS2             -0.090      0.087     -1.032      0.302

 BMI3     ON
    SSIN3             -0.038      0.071     -0.531      0.595
    SCOS3             -0.090      0.087     -1.032      0.302

 SLO      WITH
    INT                0.352      0.261      1.349      0.177

 Intercepts
    BMI1               0.000      0.000    999.000    999.000
    BMI2               0.000      0.000    999.000    999.000
    BMI3               0.000      0.000    999.000    999.000
    INT               10.672      0.919     11.616      0.000
    SLO                0.341      0.194      1.760      0.078

 Residual Variances
    BMI1              10.185      1.394      7.305      0.000
    BMI2              19.058      1.270     15.006      0.000
    BMI3               5.211      1.102      4.727      0.000
    INT                5.775      0.919      6.284      0.000
    SLO                0.399      0.120      3.331      0.001


QUALITY OF NUMERICAL RESULTS

     Condition Number for the Information Matrix              0.138E-06
       (ratio of smallest to largest eigenvalue)
```

Sensitivity analyses

Table A2 shows the results of the sensitivity analyses with regard to the procedure used to take into account incomplete data on BMI and to the combination of underweight and normal weight subjects in the same group.

The overall pattern of association and the order of magnitude of the regression coefficients is consistent across the three analyses, taking into account the reduced power of the study in the analyses B and C, due to the smaller sample size.

Variable names in the table are described in the section above (*Latent Growth Model: Mplus code and model results*).

Table S2: Parameter estimates for the regression of the slope and intercept of the growth line on subjects's baseline characteristics in the full sample (A), excluding subjects with missing values for BMI (B) and excluding underweight at baseline (C)

**Independent**	**A**		**B**		**C**	
**variable**	**Coef**	**95% CI**	**Coef**	**95% CI**	**Coef**	**95% CI**
**Slope:**						
SEX	-0.19	(-0.29 ; -0.09)	-0.21	(-0.31 ; -0.11)	-0.16	(-0.27 ; -0.05)
AGEB	-0.01	(-0.01 ; -0.01)	-0.01	(-0.01 ; -0.01)	-0.01	(-0.01 ; 0)
RACE_2	-0.05	(-0.2 ; 0.1)	-0.07	(-0.21 ; 0.08)	0.02	(-0.13 ; 0.17)
RACE_3	-0.01	(-0.18 ; 0.16)	0.01	(-0.15 ; 0.18)	-0.05	(-0.28 ; 0.19)
RACE_4	0.26	(0.05 ; 0.47)	0.27	(0.05 ; 0.49)	0.3	(0.07 ; 0.54)
EDUCAT_1	-0.02	(-0.17 ; 0.13)	-0.05	(-0.21 ; 0.11)	-0.04	(-0.21 ; 0.13)
EDUCAT_3	-0.01	(-0.11 ; 0.1)	0.02	(-0.09 ; 0.13)	-0.02	(-0.15 ; 0.11)
EDUCAT_4	0.07	(-0.08 ; 0.22)	0.09	(-0.06 ; 0.24)	0.08	(-0.09 ; 0.25)
INCQ_1	0	(-0.13 ; 0.13)	-0.01	(-0.15 ; 0.12)	0.03	(-0.12 ; 0.19)
INCQ_2	0.05	(-0.08 ; 0.19)	0.05	(-0.1 ; 0.19)	0.03	(-0.13 ; 0.18)
INCQ_4	0.13	(-0.01 ; 0.26)	0.12	(-0.02 ; 0.26)	0.13	(-0.03 ; 0.28)
INCQ_5	0.16	(0.04 ; 0.28)	0.16	(0.03 ; 0.28)	0.16	(0.02 ; 0.29)
RURAL	0.12	(0 ; 0.23)	0.1	(-0.01 ; 0.21)	0.14	(0.01 ; 0.27)
EXMOD	-0.01	(-0.15 ; 0.12)	0.02	(-0.13 ; 0.16)	-0.02	(-0.17 ; 0.12)
EXHIG	-0.15	(-0.28 ; -0.03)	-0.1	(-0.23 ; 0.03)	-0.11	(-0.28 ; 0.05)
ALC	-0.01	(-0.11 ; 0.08)	-0.03	(-0.13 ; 0.07)	-0.08	(-0.19 ; 0.02)
SMOK	-0.17	(-0.3 ; -0.04)	-0.16	(-0.3 ; -0.03)	-0.15	(-0.29 ; -0.02)
WAIST	0.01	(0 ; 0.01)	0.01	(0 ; 0.01)	0.01	(0 ; 0.01)
OVERWGT	-0.8	(-0.98 ; -0.62)	-0.73	(-0.91 ; -0.56)	-0.72	(-0.91 ; -0.53)
ORUR	-0.2	(-0.38 ; -0.02)	-0.17	(-0.35 ; 0.01)	-0.24	(-0.44 ; -0.04)
OEXMOD	0.09	(-0.14 ; 0.32)	0.06	(-0.17 ; 0.28)	0.06	(-0.17 ; 0.3)
OEXHIG	0.2	(-0.04 ; 0.44)	0.14	(-0.11 ; 0.38)	0.11	(-0.16 ; 0.37)
**Intercept:**						
SEX	-1.54	(-1.88 ; -1.21)	-1.55	(-1.9 ; -1.21)	-1.8	(-2.12 ; -1.48)
AGEB	0	(-0.02 ; 0.01)	0	(-0.02 ; 0.01)	-0.01	(-0.02 ; 0.01)
RACE_2	-0.43	(-0.9 ; 0.03)	-0.32	(-0.71 ; 0.08)	-0.63	(-1.3 ; 0.05)
RACE_3	-0.85	(-1.37 ; -0.33)	-0.74	(-1.34 ; -0.14)	-0.85	(-1.58 ; -0.12)
RACE_4	-0.35	(-1.3 ; 0.6)	-0.36	(-1.3 ; 0.58)	-0.67	(-1.78 ; 0.44)
EDUCAT_1	-0.27	(-0.75 ; 0.21)	-0.22	(-0.71 ; 0.28)	-0.08	(-0.69 ; 0.53)
EDUCAT_3	0.17	(-0.19 ; 0.53)	0.06	(-0.31 ; 0.42)	0.19	(-0.23 ; 0.6)
EDUCAT_4	0.17	(-0.38 ; 0.72)	0.01	(-0.55 ; 0.56)	0.42	(-0.18 ; 1.02)
INCQ_1	-0.18	(-0.6 ; 0.25)	-0.16	(-0.59 ; 0.27)	-0.4	(-0.92 ; 0.12)
INCQ_2	-0.16	(-0.58 ; 0.25)	-0.2	(-0.61 ; 0.2)	-0.22	(-0.7 ; 0.27)
INCQ_4	-0.26	(-0.63 ; 0.1)	-0.26	(-0.64 ; 0.13)	-0.25	(-0.67 ; 0.17)
INCQ_5	-0.21	(-0.58 ; 0.17)	-0.22	(-0.59 ; 0.16)	-0.17	(-0.6 ; 0.26)
RURAL	-0.44	(-0.78 ; -0.09)	-0.42	(-0.72 ; -0.12)	-0.46	(-0.85 ; -0.08)
EXMOD	0.35	(-0.04 ; 0.73)	0.28	(-0.09 ; 0.65)	0.41	(-0.04 ; 0.85)
EXHIG	0.75	(0.36 ; 1.15)	0.59	(0.21 ; 0.98)	0.83	(0.43 ; 1.23)
ALC	-0.22	(-0.51 ; 0.07)	-0.21	(-0.5 ; 0.09)	-0.08	(-0.41 ; 0.25)
SMOK	-0.2	(-0.6 ; 0.21)	-0.14	(-0.56 ; 0.27)	-0.35	(-0.77 ; 0.08)
WAIST	0.15	(0.13 ; 0.18)	0.15	(0.13 ; 0.18)	0.16	(0.14 ; 0.19)
OVERWGT	7.27	(6.59 ; 7.96)	6.88	(6.22 ; 7.55)	6.93	(6.23 ; 7.62)
ORUR	-0.01	(-0.6 ; 0.57)	-0.06	(-0.6 ; 0.48)	0	(-0.61 ; 0.61)
OEXMOD	-1.11	(-1.88 ; -0.33)	-1	(-1.76 ; -0.23)	-1.02	(-1.99 ; -0.05)
OEXHIG	-1.43	(-2.22 ; -0.63)	-1.24	(-2.02 ; -0.46)	-1.02	(-1.87 ; -0.17)

References

[1] Marti-Soler, H., Gubelmann, C., Aeschbacher, S., Alves, L., Bobak, M., Bongard, V., Clays, E., de Gaetano, G., Di Castelnuovo, A., Elosua, R., Ferrieres, J., Guessous, I., Igland, J., Jø rgensen, T., Nikitin, Y., O'Doherty, M.G., Palmieri, L., Ramos, R., Simons, J., Sulo, G., Vanuzzo, D., Vila, J., Barros, H., Borglykke, A., Conen, D., De Bacquer, D., Donfrancesco, C., Gaspoz, J.-M., Giampaoli, S., Giles, G.G., Iacoviello, L., Kee, F., Kubinova, R., Malyutina, S., Marrugat, J., Prescott, E., Ruidavets, J.B., Scragg, R., Simons, L.A., Tamosiunas, A., Tell, G.S., Vollenweider, P., Marques-Vidal, P., Gonseth, S., Gubelmann, C., Stringhini, S., Bovet, P., Chen, P.-C., Wojtyniak, B., Paccaud, F., Tsai, D.-H., Zdrojewski, T., Marques-Vidal, P.: Seasonality of cardiovascular risk factors: an analysis including over 230 000 participants in 15 countries. Heart **100**(19), 1517–1523 (2014). doi:10.1371/journal.pone.0113500

[2] Visscher, T.L.S., Seidell, J.C.: Time trends (1993-1997) and seasonal variation in body mass index and waist circumference in the Netherlands. Int. J. Obes. Relat. Metab. Disord. **28**(10), 1309–16 (2004). doi:10.1038/sj.ijo.0802761

[3] Cornelissen, G.: Cosinor-based rhythmometry. Theor. Biol. Med. Model. **11**, 16 (2014). doi:10.1186/1742-4682-11-16
